# Editorial: Network Bioscience

**DOI:** 10.3389/fgene.2019.01160

**Published:** 2019-11-20

**Authors:** Marco Antoniotti, Bud Mishra, Marco Pellegrini

**Affiliations:** ^1^Dipartimento di Informatica, Sistemistica e Comunicazione, Università degli Studi di Milano-Bicocca, Milan, Italy; ^2^Courant Institute of Mathematical Sciences, New York University, New York, NY, United States; ^3^Istituto di Informatica e Telematica, Consiglio Nazionale delle Ricerche, Pisa, Italy

**Keywords:** systems biology, network science, network biology, cancer networks, hypothesis generation and verification, computational biology

## Networks in Many Guises for Bioscience

In the last decade, the very nature of biological research has changed as large-scale data arrive at torrential force and it has ushered in a new era of *Bioscience*; but also this high dimensional big data is being used to support inference of various types and multiplicities of hypotheses about the extant relationships among the “variables” being measured.

The typical current example in the biomedical field is *sequencing data* (in various forms: *DNA sequencing*, *RNA sequencing*, *ATAC using sequencing*, etc.). Another kind of data currently collected is *proteomic* data, often with the goal of producing *protein-protein interaction networks* (PPI networks). Yet another is data about the *metabolome* of a biological system. Moreover recently, also phenotypic data, data on diseases, symptoms, patients, etc., are being collected at nation-wide level thus giving us another source of highly related (causal) “big data.”

From these kinds of data, biologists and bioinformaticians, can make many inferences, and, more often than not, such inferences now reuse several notions, theories, and tools from the field of *network science*. Network science has accelerated a deep and successful trend in research that influences a range of disciplines like mathematics, graph theory, physics, statistics, data science, and computer science (just to name a few), and adapts the relevant techniques and insights to address relevant but disparate social, biological, technological questions.

Most of the data kinds just mentioned naturally lend themselves to a *network* analysis. The network model is a key viewpoint leading to the uncovering of mesoscale phenomena, thus providing an essential bridge between the observable phenotypes and *omics* underlying mechanisms. Moreover, network analysis is a powerful *hypothesis generation* tool guiding the scientific cycle of *data gathering*, *data interpretation*, *hypothesis generation*, and *hypothesis testing*.

The papers contained in the present research topic—*Network Bioscience*—are examples of how network and graph analysis can be used to elucidate various aspect of biological systems from metabolic regimes, to phenotype-genotype linking, to relationships assessment among diverse omics data for therapy design, to functional submodule identification in a gene network for cancer studies.

## Papers Presentation

The papers collected in this research topic are roughly grouped as follows:

- “Foundational” papers,
- Analysis of particular biomedical problems,
- Tool presentations.

Several contributions tackle foundational aspects of network bioscience, relative to their origin, evolution, underlying philosophy, mathematical modelling, as well as connections to network medicine on one hand and dynamics of bio-chemical reactions on the other hand.

Janwa et al. explore the role of information asymmetry in the genesis and evolution of pairwise biomolecular interactions leading to the formation of extensive and complex networks of biomolecular interactions. Pusa et al. review a network-based evolutionary game-theoretic view of emerging phenotypes and its use in the context of metabolic modeling. Sonawane et al. connect the emerging field of network medicine with the opportunity of collecting big biomedical data identifying three different network archetypes according to different underlying philosophies.

Biran et al. and Nelson et al. discuss mathematical aspects of bio-networks science relative to the benefits of propagation of information in bio-networks and the benefits of embedding bio-networks into low-dimensional Euclidean spaces both for visualization and for tasks such as network de-noising, modularization, and function prediction.

Loskot et al. survey recent advances in the broad area of bio-chemical reaction networks, which constitute a crucial model for elucidating non-linear dynamics of bio-chemical processes.

Two papers report on the application of network-based models to unravel complex physiological and pathological processes, namely the molecular mechanisms causing mucociliary clearance in the human respiratory tract (Yepiskoposyan et al.) and the role of active regulatory sub-networks characterizing a genetic brain disorder: Rett Syndrome (Miller et al.).

Active subnetwork/module identification is a key step in the process of discovering differences between cases and controls (e.g., pathological and healthy states) that fully exploit the rich structure of the bio-network models, and play a key role parallel to that of DGE (differential gene expression) in comparative genomic expression analysis. Nguyen et al. contributed a review of 22 state-of-the-art integrative tools and algorithms for such problem, including a discussion of outstanding challenges and open problems. Two new original methods: *NoMAS* by Altieri et al. and *PathFindR* Ulgen et al. push forward the state of the art on active subnetwork/module identification. Tripathi et al. discuss important issues relative to benchmarking of active subnetwork/module identification methods, and to the adaptation of existing general-purpose community detection algorithms for this task.

Converting raw-data into a suitable network model is a non-trivial task, a source of very important and challenging problems. Here we have a few such examples. Tan et al. describe *QS-Net*, an accurate methodology for building phylogenetic networks from basic sequencing data. Vekris et al. develop analytical tools and strategies for de-noising phage display data, employing graph-theoretic methods. Koutsandreas et al. report on the new pipeline *ANASTASIA* for metagenomic analysis of environmental samples, which is a challenging source of data. Shafi et al. focus on the challenge of defining multi-cohort and multi-omics meta-analysis framework that overcomes limitations of less integrative approaches in order to identify robust molecular subnetworks that capture the key dynamic nature of a given biological condition. Weighill et al. unravel the multi-phenotype signatures of genes on a genome-wide network built from SNP-phenotype association data, thus improving the interpretation of large GWAS datasets and aiding in future synthetic biology efforts designed to optimize phenotypes of interest.

## Author Contributions

The editors all contributed equally to the research topic assembly and editing and to this editorial.

## Funding

This work was partially supported by the CRUK/AIRC/FC-AECC Accelerator Award #22790, *Single Cell Cancer Evolution in the Clinic*, by the FAQC 2018 *Competitive Funding Program* of the Università degli Studi di Milano-Bicocca, Milan, Italy; a National Cancer Institute Physical Sciences-Oncology Center Grant U54 CA193313-01; and by the European COST Action #CA15110 *CHARME: Harmonising standardisation strategies to increase efficiency and competitiveness of European life-science research*. This work has been partially supported within project PRIN 201534HNXC “The role of tandem repeats in neurodegenerative diseases: a genomic and proteomic approach” funded by the Italian Ministry of Education and University (MIUR) and by the National Cancer Institute Physical Sciences-Oncology Center Grant U54 CA193313-01.

## Conflict of Interest

The authors declare that the research was conducted in the absence of any commercial or financial relationships that could be construed as a potential conflict of interest.

